# Suppression of LETM1 inhibits the proliferation and stemness of colorectal cancer cells through reactive oxygen species–induced autophagy

**DOI:** 10.1111/jcmm.16169

**Published:** 2020-12-13

**Authors:** Nan Che, Zhaoting Yang, Xingzhe Liu, Mengxuan Li, Ying Feng, Chengye Zhang, Chao Li, Yan Cui, Yanhua Xuan

**Affiliations:** ^1^ Department of Pathology Yanbian University College of Medicine Yanji China; ^2^ Institute for Regenerative Medicine Yanbian University College of Medicine Yanji China; ^3^ Department of Oncology Affiliated Hospital of Yanbian University Yanji China

**Keywords:** autophagy, colorectal cancer, LETM1, reactive oxygen species

## Abstract

Leucine zipper‐EF‐hand–containing transmembrane protein 1 (LETM1) is a mitochondrial inner membrane protein that is highly expressed in various cancers. Although LETM1 is known to be associated with poor prognosis in colorectal cancer (CRC), its roles in autophagic cell death in CRC have not been explored. In this study, we examined the mechanisms through which LETM1 mediates autophagy in CRC. Our results showed that LETM1 was highly expressed in CRC tissues and that down‐regulation of LETM1 inhibited cell proliferation and induced S‐phase arrest. LETM1 silencing also suppressed cancer stem cell–like properties and induced autophagy in CRC cells. Additionally, the autophagy inhibitor 3‐methyladenine reversed the inhibitory effects of LETM1 silencing on proliferation and stemness, whereas the autophagy activator rapamycin had the opposite effects. Mechanistically, suppression of LETM1 increased the levels of reactive oxygen species (ROS) and mitochondrial ROS by regulation of SOD2, which in turn activated AMP‐activated protein kinase (AMPK)/mammalian target of rapamycin (mTOR), initiated autophagy, and inhibited proliferation and stemness. Our findings suggest that silencing LETM1 induced autophagy in CRC cells by triggering ROS‐mediated AMPK/mTOR signalling, thus blocking CRC progression, which will enhance our understanding of the molecular mechanism of LETM1 in CRC.

## 1 INTRODUCTION

Colorectal cancer (CRC) is the third most common cancer in men and the second most common cancer in women worldwide.^1^ More than 1.8 million new CRC cases and 881,000 deaths related to CRC were reported in 2018, accounting for approximately 1 in 10 cancer cases and deaths.^2^ Despite the success of screening programmes and the development of adjuvant therapies, the global burden of CRC is projected to increase by 60% to more than 2.2 million new cases and 1.1 million deaths by 2030.^3^ Thus, new molecular targets for therapeutic intervention are urgently required.

Autophagy is a cellular degradation process that recycles or degrades internal constituents through a membrane‐trafficking pathway to sustain the homeostasis of normal cells.^4^ The functions of autophagy in tumour cells are complex and may either promote or inhibit proliferation through different signalling contexts under specific conditions in diverse cancers.^5^, ^6^, ^7^ For example, autophagy is a principle cause of drug resistance in cancer cells during chemotherapy. However, excessive autophagy can result in autophagic cell death and inhibit the occurrence and progression of malignancy.^8^ Thus, the extent of autophagy determines the survival or death of cells. At low concentrations, reactive oxygen species (ROS), which are products of cellular metabolism, can promote cell growth and proliferation by regulating diverse signalling pathways as secondary messengers.^9^, ^10^ In contrast, high ROS concentrations result in cell death by activating apoptosis^11^ or autophagy.^12^ Previous studies have shown that many drugs exert anticancer effects by stimulating ROS production to induce apoptosis and autophagic cell death.^13^

Leucine zipper‐EF‐hand–containing transmembrane protein 1 (LETM1) is a mitochondrial inner membrane protein and has been proposed as a Ca^2+^/H^+^ exchanger which can mediate the rate of uptake and excretion of mitochondrial Ca^2+^ in a concentration‐dependent manner.^14^, ^15^ In addition, others submitted that LETM1 plays an imperative role in mitochondrial K^+^ homeostasis by mediating the mitochondrial K^+^/H^+^ exchange.^16^ In this respect, loss of LETM1 results in mutations of mitochondrial bioenergetics and metabolic signalling, and even cell death.^14^, ^17^ Moreover, LETM1 is highly expressed in many human malignant tumours and is closely associated with lymph node metastasis, disease‐free survival and overall survival rates.^18^, ^19^, ^20^ LETM1 expression has been detected in CRC tissues and is associated with the prognosis of patients with colorectal adenocarcinoma.^21^ However, the roles and molecular mechanisms of LETM1 in CRC cell autophagy remain unclear.

In this study, we investigated whether LETM1 down‐regulation stimulates the production of ROS to activate the AMP‐activated protein kinase (AMPK)/mammalian target of rapamycin (mTOR) signalling pathway and autophagy, eventually leading to inhibition of the proliferation and stemness of CRC cells.

## 2 MATERIALS AND METHODS

### 2.1 Cell culture

The HT29 and HCT116 cell lines were purchased from the American Type Culture Collection and grown in the RPMI‐1640 culture medium (Life Technologies) containing 10% foetal bovine serum (Life Technologies), 100 mg/mL penicillin and 50 mg/mL streptomycin (Life Technologies) in 5% humidified CO_2_ incubator at 37°C.

### 2.2 RNA interference

HT29 and HCT116 cells were transfected with endo‐ribonuclease prepared siRNA (esiRNA) targeting LETM1 (Sigma‐Aldrich). The cells were cultured with serum‐free medium for 24 hours in 6‐well plates. Transfection was performed by Lipofectamine 3000 (Invitrogen, Life Technologies) according to the manufacturer's instructions. The sequence of LETM1 esiRNA is listed in Table S1.

### 2.3 Colony formation assay

200 cells/well were cultured in 6‐well plates for 10 days until macroscopic colonies appeared. After washed and fixed, the cells were stained with Giemsa (Solarbio). Colonies with a diameter >1 mm were counted.

### 2.4 Carboxyfluorescein diacetate succinimidyl ester (CFSE) cell proliferation assay

CellTraceTM CFSE (Invitrogen) staining was performed to measure cell proliferation. The CRC cell suspensions of 10^6^ cells/mL were labelled with 2 μmol/L CFSE at 37°C for 20 minutes. Then, stained cells were cultured with RPMI‐1640 (containing 10% FBS) for 5 minutes to terminate reaction. After washing with RPMI‐1640, CFSE‐labelled cells were seeded into 6‐well plates and grown at 37°C and 5% CO_2_ for 3 days. Flow cytometer (Beckman Coulter) was used to read samples at a wavelength of 488nm. The decreased fluorescent intensity indicates a high rate of cell proliferation.

### 2.5 Cell cycle and cell death analysis

Cells were collected and fixed in cold 70% ethanol at −20°C overnight and then incubated in 10 µg/mL propidium iodide (PI) solution containing 5 µg/mL RNase A (BD Biosciences) for 30 minutes at RT in the dark. For the cell death analysis, cells were collected and incubated with 5 μg/mL PI for 1 minute. The results were detected with a flow cytometer at 488 nm wavelengths.

### 2.6 Immunofluorescence assay and Western blotting

Immunofluorescence (IF) and Western blotting (WB) procedures were performed according to previously described protocols.^22^ Antibodies used in present study are listed in Table S2. β‐Actin and GAPDH were used as the loading control.

### 2.7 Sphere formation assay

3000 cells/well were cultured in DMEM/F12 (Invitrogen) supplemented with 2% B27 (Invitrogen), 20 ng/mL EGF (Invitrogen) and 20 ng/mL FGF‐b (Invitrogen). Sphere formation efficiency was determined as follows: sphere/3000 cells × 100%.

### 2.8 Monodansylcadaverine (MDC) staining

Monodansylcadaverine staining (Solarbio) was performed to analyse autophagic process. After transfection with esi‐LETM1, cells were incubated with 100μl wash buffer containing 10 μL MDC staining for 30 minutes in the dark. The results were observed by Cytation 5 imaging reader (BioTek).

### 2.9 Transmission electronic microscope (TEM)

After 24‐h esi‐LETM1 transfection, CRC cells were harvested and fixed with glutaraldehyde and OsO4, respectively. After being dehydrated with a gradient alcohol, the cells were embedded in epoxy resin and sectioned. Afterwards, the ultrathin sections were stained by lead citrate and uranyl acetate. Autophagosomes in these cells were observed with TEM.

### 2.10 Detection of ROS

Reactive Oxygen Species Assay Kit (Beyotime Biotechnology, Shanghai, CN) containing DCFH‐DA was used to detect intracellular ROS. Cells were incubated with 10 μmol/L DCFH‐DA for 30 minutes at 37°C in accordance with the manufacturer's instructions. The fluorescence intensity was measured by Cytation 5 imaging reader or flow cytometer.

### 2.11 Determination of mROS

CRC cells were seeded in 6‐well plates with coverslips and loaded with the mitochondrial superoxide indicator MitoSOX Red (Invitrogen) for 30 minutes at 37°C. Hoechst 33342 (Beyotime Biotechnology) was used as nuclear counterstain. Images were acquired using Cytation 5 imaging reader.

### 2.12 Databases analysis

*Oncomine* database (www.oncomine.org) was used to check the expression values of LETM1 in normal colon tissues and CRC tissues. We also used data set GSE3494212 obtaining from the Gene Expression Omnibus (GEO) database (www.ncbi.nlm.nih.gov/geo/) to identify gene sets correlated with LETM1 by gene set enrichment analysis (GSEA), including three key statistics: false discovery rate (FDR), normalized enrichment score (NES) and nominal p‐value. Then, Gene Expression Profiling Interactive Analysis (GEPIA) database (http://gepia.cancer-pku.cn) and cBioPortal for Cancer Genomics tools (http://www.cbioportal.org/) were used for validating pairwise gene correlation by the Pearson correlation statistics.

### 2.13 Statistical analysis

Statistical analysis was performed with the GraphPad Prism software (version 7.00; GraphPad Prism Software, Inc). Statistically significant differences between groups were calculated using two‐tailed paired Student's *t* test or one‐way ANOVA. All values were expressed as the mean ± standard deviation from three independent experiments. Asterisks represent the degree of significance: *P*‐ values: n.s = *P* ≥ .05, *P < .05, **P < .01, ***P < .001 and ****P < .0001.

## 3 RESULTS

### 3.1 Silencing of LETM1 suppressed CRC cell proliferation and stemness

**FIGURE 1:**
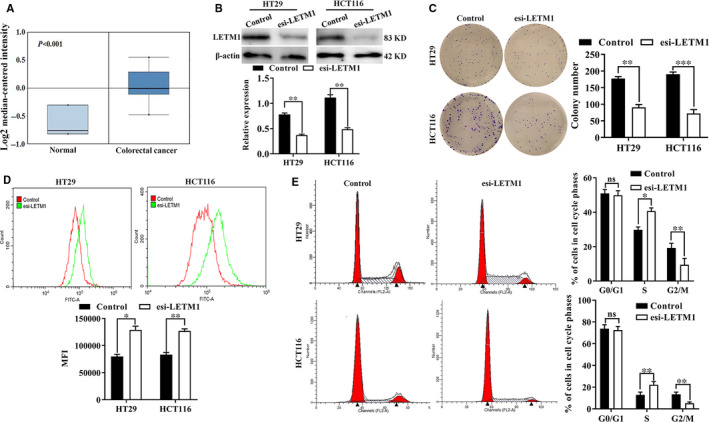
LETM1 was up‐regulated in CRC tissues, and silencing of LETM1 suppressed the proliferation of CRC cells. A, *LETM1* mRNA expression in CRC tissues and normal colon tissues from the Oncomine database. B, Silencing of LETM1 in HT29 and HCT116 cells was confirmed by WB. After transfection with esi‐LETM1, cell proliferation was evaluated by colony formation assays (C) and CFSE staining (D). E, Cell cycle distribution in HT29 and HCT116 cells transfected with esi‐LETM1, as determined by flow cytometry. The bar graph shows the relative cell populations in G_0_/G_1_, S and G_2_/M phases

First, we analysed the gene expression profile of LETM1 in CRC using the online microarray database Oncomine. The results indicated that LETM1 was overexpressed in CRC tissues compared with that in normal colon tissues (Figure 1A), consistent with the results of a previous study.^21^ We used specific esiRNA to establish LETM1 functional expression in HT29 and HCT116 cells and confirmed the efficiency of LETM1 silencing by WB (Figure 1B). Next, colony and CFSE assays were utilized to investigate the roles of LETM1 in CRC cell proliferation. The results suggested that knockdown of LETM1 significantly decreased colony formation efficiency and proliferation capacity (Figure 1C,D). These results suggested that LETM1 promoted CRC cell proliferation.

To investigate the involvement of LETM1 in CRC cell proliferation, we next examined the cell cycle distribution. Notably, LETM1‐silenced CRC cells showed decreased G_2_/M‐phase subpopulations and accumulation of S‐phase cells but no significant changes in G_0_/G_1_‐phase subpopulations compared with controls (Figure 1E). Analysis of the expression of key genes involved in the S‐to‐G_2_ phase transition demonstrated that LETM1 mRNA expression was positively correlated with cyclin A2 and cyclin‐dependent kinase (CDK) 2 in CRC samples (Figure S1A,B), consistent with the analysis of cBioPortal (Figure S1C,D). In addition, the results of IF revealed that LETM1 co‐localized with cyclin A2 and CDK2 in colorectal cancer cells (Figure S1E,F). Further analysis of the GEO database (tumours from 17 patients with CRC) using GSEA showed that positively regulated genes related to G_2_/M phase were enriched in the LETM1‐high expression group (NES = 1.5126858, FDR *q*‐value = 0.17041634; Figure S1G). Taken together, these data indicated that LETM1 promoted cell proliferation mainly through modulating cell cycle progression.

Cancer stem–like cells (CSCs) comprise a small fraction of malignant cells and are responsible for cancer proliferation because of their capacity for self‐renewal.^23^ Previous studies have shown that high LETM1 level is closely related to cancer stemness proteins in CRC.^21^ Similarly, in this study, IF assays demonstrated that LETM1 was co‐expressed with CSC markers (CD44 and CD133) in CRC tissues (Figure S2A). Accordingly, we next examined the regulatory impact of LETM1 on cancer stemness characteristics in CRC cells. First, we determined CD44 and CD133 protein levels after blocking LETM1. The results showed that suppression of LETM1 in CRC cells led to significant reductions in levels of CD44 and CD133 (Figure S2B). To further determine whether LETM1 contributed to the stemness properties of CRC cells, we performed sphere formation assays following transfection with or without esi‐LETM1. In the presence of esi‐LETM1, the size and number of spheres were significantly decreased compared with that in controls (Figure S2C). Moreover, IF assay results showed that transfection with LETM1 esiRNA significantly reduced the levels of CD44 and CD133 in CRC spheroid cells (Figure S2D). In summary, these observations demonstrated that down‐regulation of LETM1 inhibited the stemness of CRC cells.

### 3.2 Inhibition of LETM1 activated autophagy in CRC cells

**FIGURE 2:**
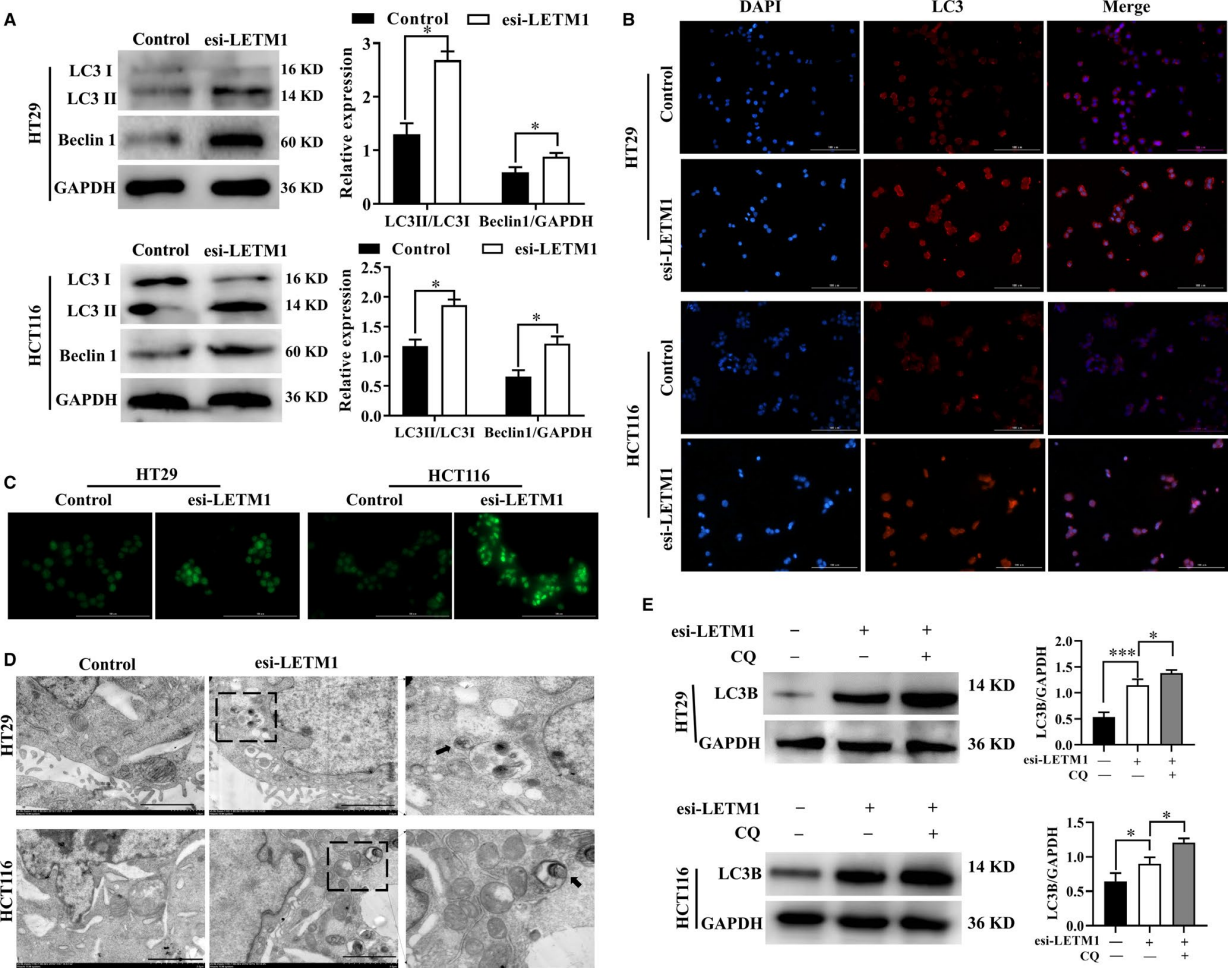
Inhibition of LETM1 activated autophagy in CRC cells. A, WB was performed to assess the protein levels of LC3 and Beclin1 in LETM1‐silenced HT29 and HCT116 cells. B, Protein levels of LC3 in HT29 and HCT116 cells in the presence or absence of esi‐LETM1 were observed by IF. Cells were counterstained with Hoechst 33342. Scale bar, 100 μm. C, MDC was performed to observe autophagolysosomes in CRC cells. Scale bar, 100 μm. D, Cells were transfected with esi‐LETM1 for 24 h and prepared for TEM analysis. The autophagosomes were marked with red arrows. E, Cells were transfected with esi‐LETM1 either alone or in the presence of CQ (50 μmol/L) and autophagy flux was monitored by WB analysis

Because autophagy is often associated with cancer cell growth and death, we tested the effects of LETM1 on autophagy in CRC cells. At 24 hours after transfection with esi‐LETM1, WB was conducted to detect Beclin1 and LC3, which are key protein markers of autophagy activation.^24^ As shown in Figure 2A, transfection with esi‐LETM1 caused significant increases in Beclin1 protein level and the LC3II/I ratio in CRC cells. Moreover, IF staining also showed that LC3 level in LETM1‐silenced cells was additionally increased compared with that in control cells (Figure 2B). To further characterize the functional roles of LETM1 in autophagy, MDC and IF staining were performed in cultured CRC cells. MDC can be used to preferentially label autophagosomes via its integration into lipids in autophagic vacuoles.^25^ Blocking of LETM1 markedly elevated autophagic vacuole formation (Figure 2C). Then, TEM was performed to further examine the autophagosomes of LETM1‐silenced cells. As shown in Figure 2D, abnormal formation and accumulation of vesicles with double‐membrane structures were observed after cells were transfected with esi‐LETM1. Moreover, to monitor intracellular autophagic flux after silencing LETM1, we examined LC3 alterations after chloroquine (CQ) treatment. The results showed that CQ treatment increased LC3 level, indicating enhanced autophagy flux in CRC cells during esi‐LETM1 transfection (Figure 2E). Collectively, our data supported that inhibition of LETM1 induced autophagy in CRC cells.

### 3.3 Down‐regulation of LETM1 inhibited proliferation and stemness through autophagy in CRC cells

**FIGURE 3:**
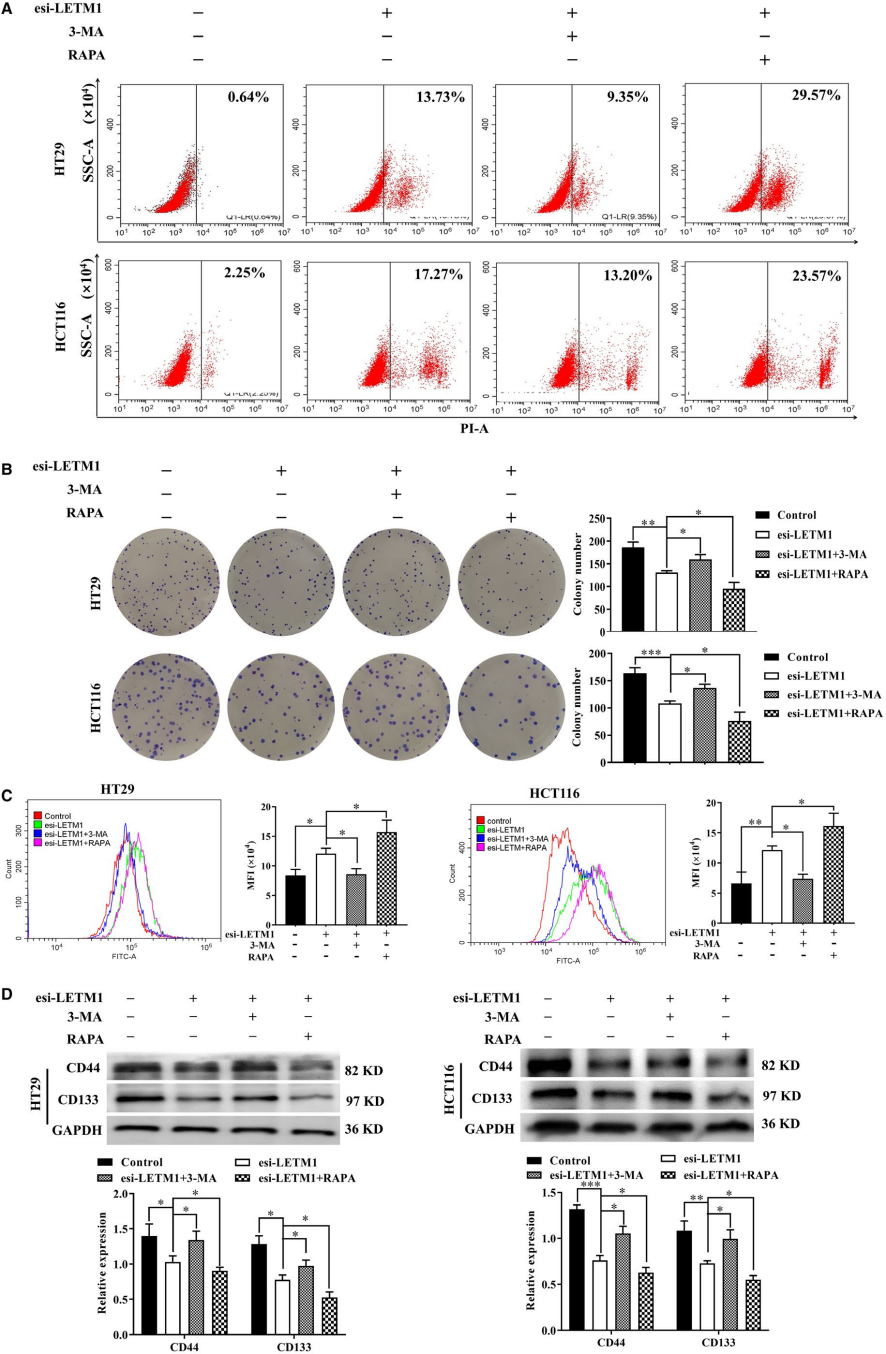
Down‐regulation of LETM1 inhibited proliferation and stemness through autophagic cell death in CRC cells. HT29 and HCT116 cells transfected with esi‐LETM1 were treated with autophagy inhibitor 3‐MA (2 mmol/L) or activator RAPA (5 μmol/L) for 24 h. A, Flow cytometry was used to detect cell death. Colony formation (B) and CFSE assays (C) were performed to determine proliferation. D, WB was used to analyse the level of CD44 and CD133

Because LETM1 promoted the proliferation and stemness of CRC cells, we next explored the underlying mechanisms. Although LETM1 has been linked to autophagy and was recently revealed as a key player in CRC development and progression, no studies have determined the mechanisms through which LETM1‐mediated autophagy regulates proliferation and stemness in CRC cells. Therefore, to examine whether autophagy was involved in LETM1‐dependent proliferation and stemness in CRC cells, we treated the cells with the autophagy inhibitor 3‐methyladenine (3‐MA) or the autophagy activator rapamycin (RAPA) for 24 hours after transfection and evaluated cell death and proliferation. As shown in Figure S3A,B, 3‐MA or RAPA alone had no significant effect on cell death or proliferation. However, down‐regulation of LETM1 dramatically enhanced cell death; this effect was blocked by 3‐MA cotreatment and enhanced by RAPA treatment (Figure 3A), indicating that LETM1 knockdown induced autophagic cell death. Moreover, colony formation and CFSE assays indicated that treatment with 3‐MA reversed the inhibitory effects of LETM1 knockdown on cell proliferation, whereas RAPA enhanced these effects (Figure 3B,C). In the presence of esi‐LETM1, 3‐MA elevated CD44 and CD133 expression, whereas RAPA had the opposite effect (Figure 3D). Therefore, silencing of LETM1 induced autophagic cell death, which contributed to inhibition of the proliferation and stemness of CRC cells.

### 3.4 Silencing of LETM1 promoted autophagy via the AMPK/mTOR signalling pathway in CRC cells

**FIGURE 4:**
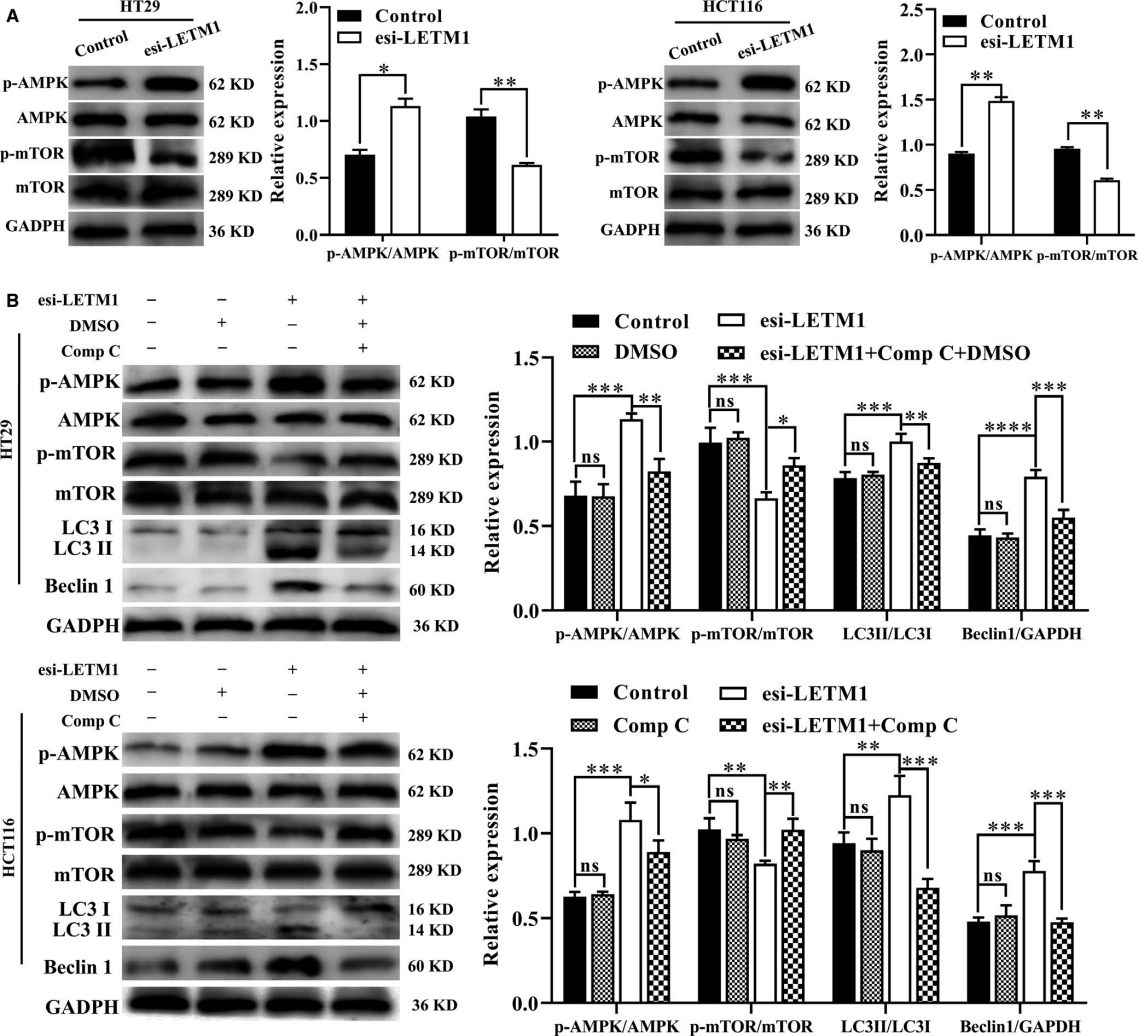
Silencing of LETM1 promoted autophagy via the AMPK/mTOR signalling pathway in CRC cells. A, After transfection with esi‐LETM1 for 24 h, WB was performed to detect AMPK, phospho‐AMPK, mTOR and phospho‐mTOR. B, HT29 and HCT116 cells were transfected with esi‐LETM1 and then treated with AMPK inhibitor Comp C (5 μmol/L) dissolved in DMSO for 24 h. WB was performed to determine AMPK, phospho‐AMPK, mTOR, phospho‐mTOR, LC3 and Beclin1 levels

Recently, the AMPK/mTOR signalling pathway has been shown to play major roles in cell autophagy; specifically, AMPK activation dephosphorylates mTOR, resulting in initiation of autophagy.^26^ Additionally, LETM1 has been shown to regulate AMPK in HeLa cells.^27^ Therefore, in this study, we further investigated the effects of LETM1 silencing on AMPK/mTOR activity in CRC cells. As expected, down‐regulation of LETM1 enhanced AMPK phosphorylation and reduced mTOR phosphorylation in both cell lines (Figure 4A). Analysis of the GEPIA databases showed that LETM1 expression was significantly correlated with mTOR in CRC tissues (Figure S4). Therefore, these results indicated that LETM1 silencing triggered the AMPK/mTOR pathway.

Next, to determine whether AMPK/mTOR activation was involved in LETM1 silencing‐induced autophagy, the AMPK inhibitor compound C (Comp C) was used to inhibit AMPK/mTOR. Indeed, Comp C decreased phospho‐AMPK levels and increased phospho‐mTOR levels in LETM1‐silenced CRC cells (Figure 4B). We then evaluated the protein levels of autophagy‐related proteins by WB after Comp C treatment in LETM1‐silenced CRC cells. As shown in Figure 4B, the stimulatory effects of LETM1 knockdown on Beclin1 and LC3 levels were blocked by Comp C, indicating that down‐regulation of LETM1‐induced autophagy was mediated by the AMPK/mTOR pathway.

### 3.5 Suppression of LETM1 induced autophagy via the ROS‐mediated AMPK/mTOR signalling pathway in CRC cells

**FIGURE 5:**
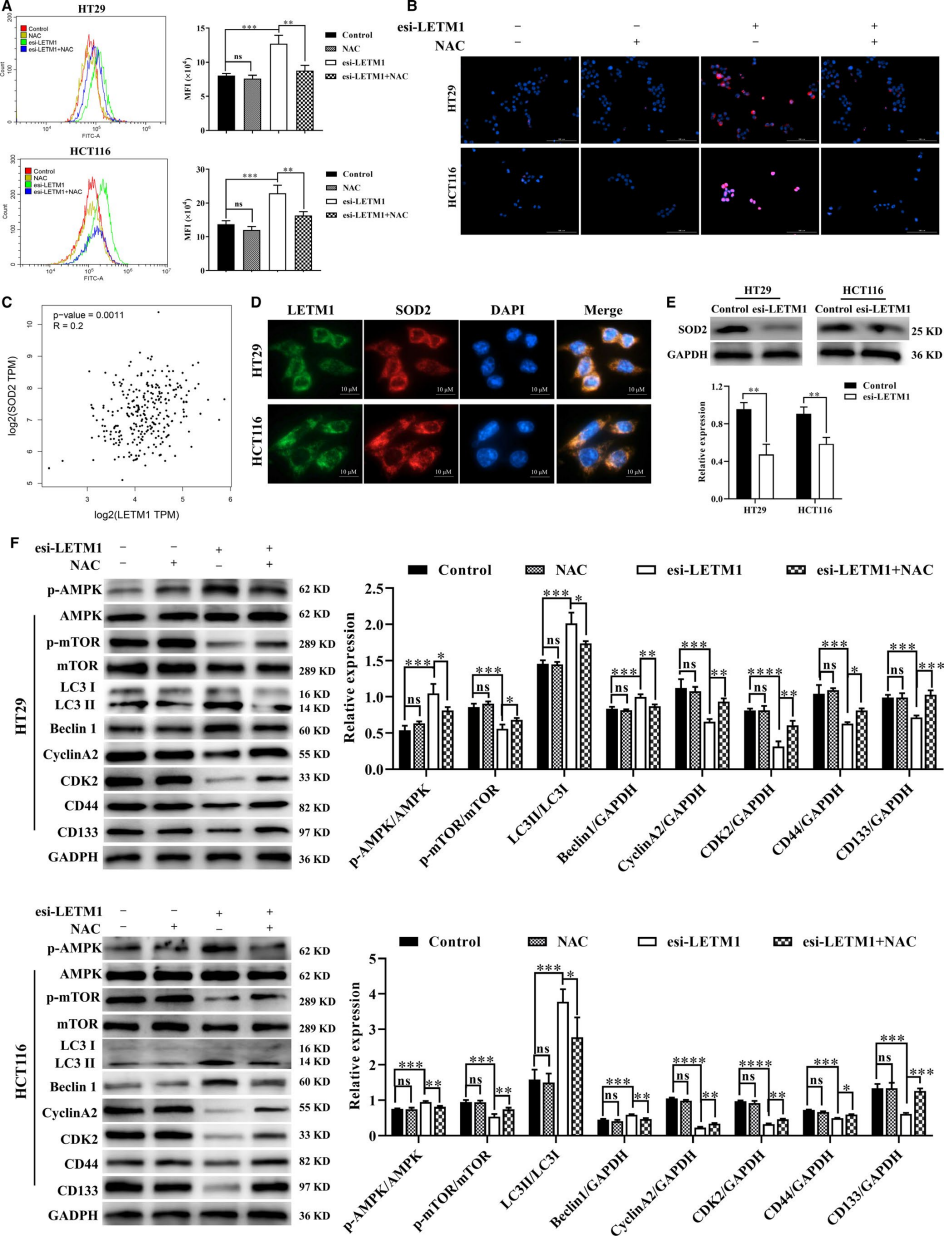
Suppression of LETM1 induced autophagy via the ROS‐mediated AMPK/mTOR signalling pathway in CRC cells. HT29 and HCT116 cells were transfected with esi‐LETM1 and treated with ROS scavenger NAC (10 μmol/L) for 24 h. A, DCFH‐DA was used to detect cellular ROS levels by flow cytometry. B, MitoSOX Red was used to detect mROS. Cells were counterstained with Hoechst 33342. Scale bar, 100 μm. C, Correlations between LETM1 and SOD2 in CRC tissues in TCGA databases using GEPIA. D, Co‐expression of LETM1 with SOD2 in CRC cells by IF. E, SOD2 protein levels were detected in CRC cells with or without esi‐LETM1 by WB. F, The protein levels of AMPK, phospho‐AMPK, mTOR, phospho‐mTOR, LC3, Beclin1, cyclin A2, CDK2, CD44 and CD133 were then evaluated using WB

ROS, mainly produced by mitochondria, are involved in many cellular functions. Thus, we investigated the relationship between LEMT1, a mitochondrial inner membrane protein, and cellular ROS. As shown in Figure 5A,B and Figure S5A, DCFH‐DA and MitoSOX Red staining after transfection of cells with esi‐LETM1 revealed that LETM1 silencing increased ROS and mROS levels in CRC cells. These effects were reversed by the ROS scavenger *N*‐acetyl‐l‐cysteine (NAC). Because ROS levels are regulated by multiple antioxidant enzymes, including Mn‐containing SOD2 in the mitochondrial matrix,^28^ we first analysed TCGA database by GEPIA and explored the correlations between LETM1 and SOD2 in CRC tumours. Interestingly, there was a significant positive relationship between the expression of LETM1 and SOD2 (Figure 5C), which was consistent with the results analysed by cBioPortal (Figure S5B). Furthermore, LETM1 was co‐localized with SOD2 in CRC cells (Figure 5D). In parallel, WB results confirmed that SOD2 levels were down‐regulated by knocking down LETM1 in CRC cells (Figure 5E). Taken together, these results showed that down‐regulation of LETM1 may promote ROS production by regulating SOD2 in CRC cells.

Previous studies have demonstrated that ROS activates the AMPK signalling pathway by boost energy consumption, resulting in initiation of autophagy.^29^, ^30^ Because our results showed that LETM1 mediated the AMPK/mTOR signalling pathway, we evaluated the roles of ROS in LETM1 silencing‐dependent AMPK/mTOR activation. The results showed that NAC decreased phospho‐AMPK levels and increased phospho‐mTOR levels (Figure 5F). Further analysis of autophagy‐related protein levels by WB and IF staining after cotreatment with NAC and LETM1 esiRNA indicated that NAC decreased the levels of Beclin1 and LC3 in LETM1‐silenced CRC cells (Figure 5F; Figure S5C). Furthermore, cotreatment with NAC significantly increased the levels of proliferation‐related proteins (cyclin A2 and CDK2) and stemness‐related proteins (CD44 and CD133; Figure 5F). In additionally, NAC could prevent cell death induced by silencing LETM1 (Figure S5D). Therefore, these data demonstrated that silencing of LETM1 activated ROS production and induced AMPK/mTOR‐mediated autophagy, which may have inhibited the proliferation and stemness of CRC cells.

## 4 DISCUSSION

LETM1 is an inner mitochondrial membrane protein that plays critical roles in mitochondrial ion homeostasis and cell viability.^31^ Recent studies have shown that LETM1 also has important roles in tumorigenesis and tumour development. For example, Huang et al^18^ reported that LETM1 promotes the proliferation and invasion of bladder cancer cells. Here, we explored the functions of LETM1 in the proliferation and stemness of CRC cells by genetic silencing of LETM1 expression. We found that LETM1 had potent stimulatory effects on CRC. Down‐regulation of LETM1 also inhibited the growth of CRC cells by suppressing cell proliferation and stemness. Interestingly, LETM1 was found to be a potent autophagy regulator in CRC cells, and LETM1 silencing initiated autophagy by activating the ROS/AMPK/mTOR signalling pathway.

Analysis of the Oncomine database revealed that LETM1 expression was significantly elevated in CRC tissues, which is consistent with the findings of a previous study.^21^ Thus, LETM1 may promote the progression of colorectal tumours. Indeed, a study by Huang et al^18^ showed that suppression of LETM1 inhibited the proliferation of bladder cancer cells, consistent with our results demonstrating that LETM1 down‐regulation blocked CRC cell proliferation. Furthermore, silencing of LETM1 caused S‐phase arrest, similar to the results of Doonan et al,^27^ who showed that knockdown of LETM1 induced aberrant accumulation of S‐phase cells, which could be reversed by re‐expression of LETM1. CSCs mediate tumour development, growth, regeneration and self‐renewal.^32^ Many stem cell markers, such as CD44 and CD133, have critical roles in the formation and development of tumours.^33^, ^34^ Recent reports suggest that LETM1 is associated with cancer stem cell–like properties.^35^ Herein, we found that LETM1 silencing resulted in down‐regulation of the CSC‐associated proteins CD44 and CD133 and decreased sphere‐forming ability. Our results confirmed the findings of Piao^21^ and showed that LETM1 is an important factor associated with cancer stemness in CRC. These findings support the crucial roles of LETM1 in the proliferation and stemness of CRC cells.

Autophagy is an evolutionarily conserved degradation system that degrades damaged organelles or misfolded proteins inside of cells and is associated with human diseases and physiologies, including cancer, genomic damage and metabolic stress.^5^, ^36^ Autophagy is generally thought to play a role in promoting cell survival. However, recent studies have shown that autophagy exerts cytotoxic effects by inducing cell death in several cancers, suggesting potential anticancer applications.^37^ Knockdown of LETM1 induces autophagy in HeLa cells.^27^ In the current study, we confirmed the effects of LETM1 silencing on autophagy in CRC cells, leading to robust accumulation of autophagolysosomes and significant enhancement of the levels of autophagic key proteins. The autophagy inhibitor 3‑MA significantly reduced the inhibitory effects of LETM1 silencing on proliferation and stemness, whereas the autophagy activator RAPA enhanced these effects. Moreover, autophagy induced by LETM1 silencing further promoted cell death in CRC cells. Overall, these results suggested that LETM1 knockdown negatively regulated proliferation and stemness by inducing autophagic cell death in CRC cells.

To date, several cellular signalling pathways have been implicated in the initiation of autophagy. Among them, the AMPK/mTOR pathway has attracted wide attention. AMPK serves as a positive regulator of autophagy by indirectly or directly suppressing the activity of mTOR, which acts to inhibit autophagy. Knockdown of LETM1 results in aberrant mitochondrial Ca^2+^ uptake, which decreases ATP levels.^27^ These effects may lead to AMPK activation and subsequently trigger autophagy initiation. Our study indicated that LETM1 silencing markedly increased phospho‐AMPK levels and decreased phospho‐mTOR levels, subsequently activating autophagy in CRC cells.

The main sources of cellular ROS are mitochondrial respiration, peroxisomal β‐oxidation, NADPH oxidases and the endoplasmic reticulum.^38^ Under normal conditions, ROS can act as signals to regulate cell proliferation and survival; however, excessive accumulation of ROS plays a significant role in cell death by controlling various signalling pathways, which are closely related to cancer progression.^39^, ^40^ In the current study, we found that inhibition of LETM1 promoted ROS and mROS generation, which is consistent with the findings of Doonan's study.^27^ SOD2 has been identified as an antioxidant enzyme that regulates ROS levels in the mitochondrial matrix. Targeting the SOD2/ROS signal pathway can inhibit NSCLC progression.^41^ Accordingly, we successfully proved that SOD2 levels were decreased in LETM1‐knockdown CRC cells, suggesting that down‐regulation of LETM1 may promote ROS production by inhibiting SOD2. In addition, ROS regulate phospho‐AMPK levels and have crucial roles in induction of AMPK‐dependent autophagy.^42^ Furthermore, the ROS scavenger NAC abrogates the inhibitory effects of down‐regulated LETM1 on cell autophagy and AMPK/mTOR, suggesting that knockdown of LETM1 inhibited cell proliferation and stemness by inducing ROS/AMPK/mTOR–mediated autophagy.

## 5 CONCLUSION

**FIGURE 6:**
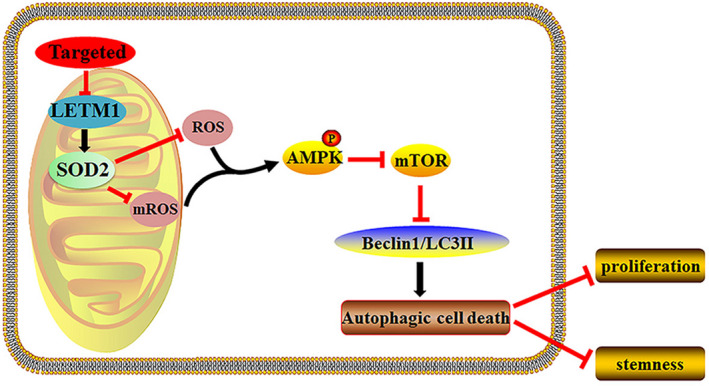
Mechanisms of LETM1 down‐regulation‐induced autophagy in CRC cells. LETM1 silencing stimulates the production of cellular ROS and mROS to activate the AMPK/mTOR signalling pathway, which then initiates autophagy and eventually inhibits the proliferation and stemness of CRC cells

In summary, our findings suggested that suppression of LETM1 enhanced ROS generation in CRC cells, thereby inhibiting proliferation and stemness via AMPK/mTOR–regulated autophagy (Figure 6). Our results provided insights into the potential mechanisms through which LETM1 mediates autophagy in CRC cells and LETM1 may participate in intracellular redox balance by regulating SOD2. However, whether LETM1 would affect tumour cell metabolism through mitochondrial oxidative phosphorylation has not been investigated. Addressing such limitations can serve as a basis for conducting future studies.

## CONFLICT OF INTEREST

All the authors declare that there is no conflict of interest.

## AUTHOR CONTRIBUTION

**Nan Che** **:** Conceptualization (lead); Formal analysis (equal); Investigation (equal); Methodology (lead); Writing‐original draft (lead); Writing‐review & editing (equal). **Zhaoting Yang:** Conceptualization (equal); Validation (equal); Writing‐original draft (supporting). **Xingzhe Liu:** Data curation (equal); Investigation (equal). **Mengxuan Li:** Data curation (equal); Software (equal). **Ying Feng:** Data curation (equal); Investigation (equal). **Chengye Zhang:** Formal analysis (equal). **Chao Li:** Formal analysis (equal). **Yan Cui:** Conceptualization (equal); Funding acquisition (equal); Methodology (equal); Writing‐review & editing (equal). **Yanhua Xuan:** Funding acquisition (lead); Project administration (lead); Resources (lead); Supervision (lead); Writing‐review & editing (equal).

## Supporting information

Fig S1

Fig S2

Fig S3

Fig S4

Fig S5

Tab S1‐S2
