# Supplementary figures and images for: Suppression of LETM1 inhibits the proliferation and stemness of colorectal cancer cells through reactive oxygen species–induced autophagy
Source: J Cell Mol Med. 2020 Dec 13;25(4):2110–20. doi: 10.1111/jcmm.16169 (PMC7882971; doi:10.1111/jcmm.16169)

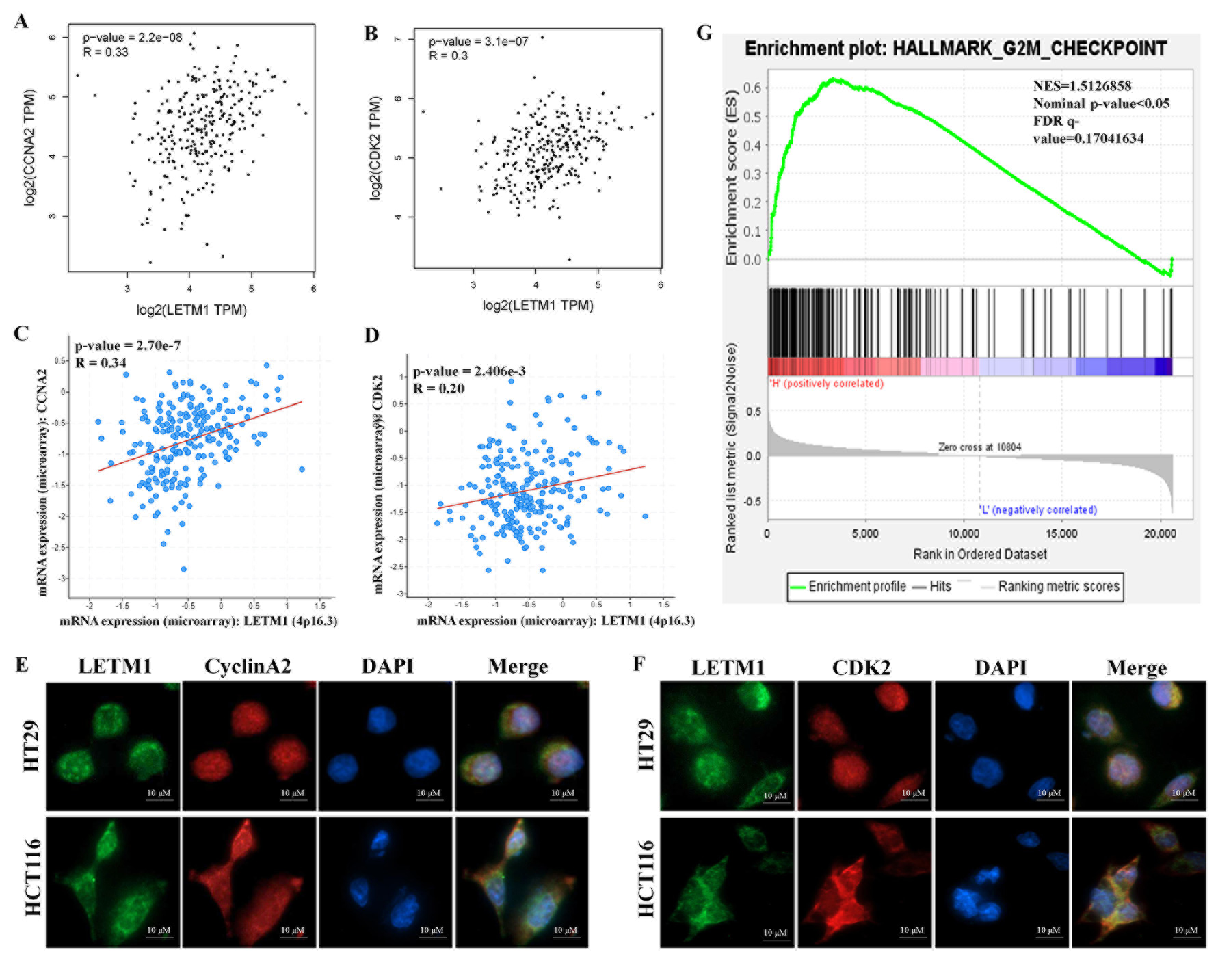

Supplement: Supplementary file 1 — Fig S1 [file JCMM-25-2110-s001.tif]

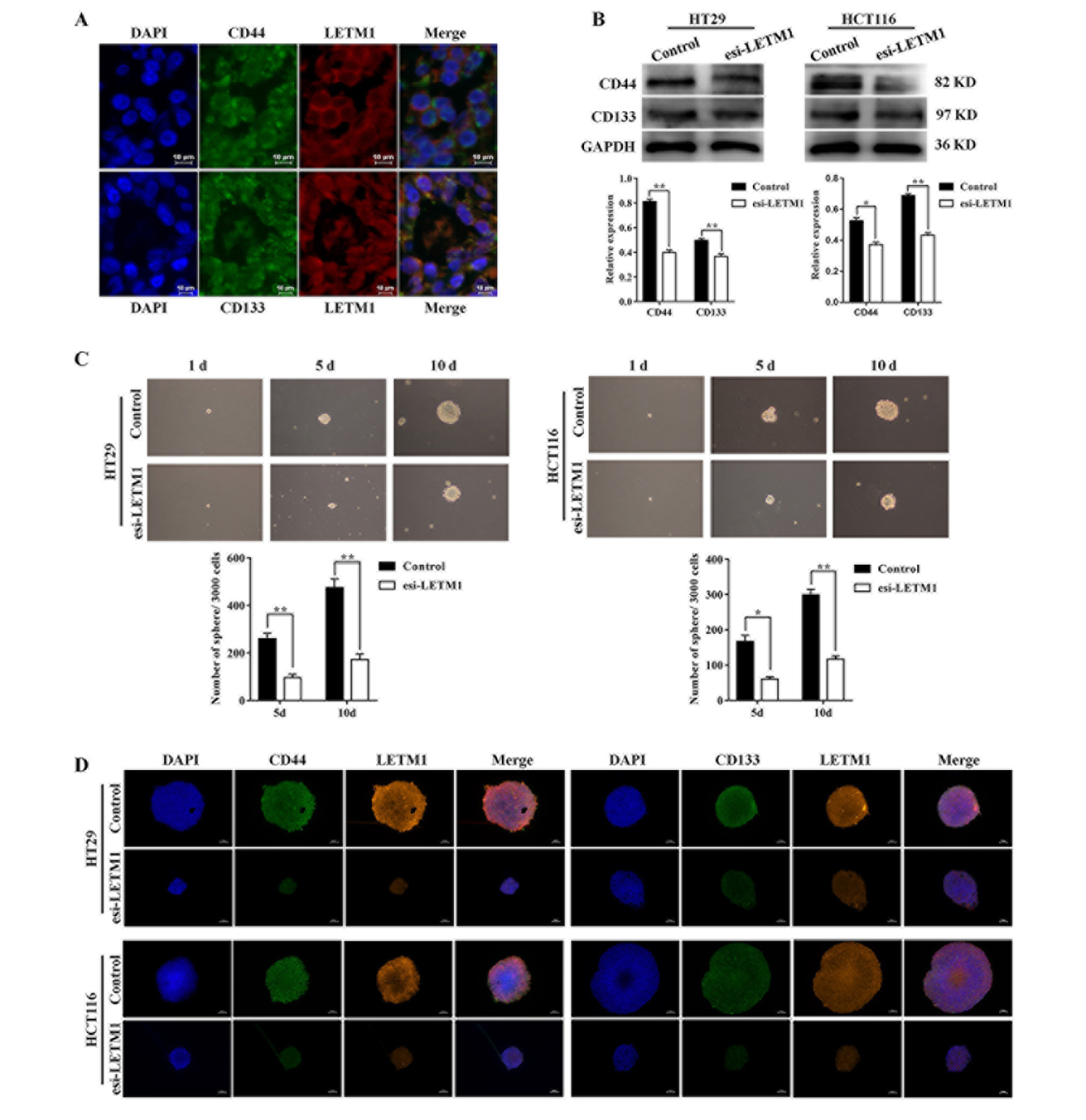

Supplement: Supplementary file 2 — Fig S2 [file JCMM-25-2110-s002.tif]

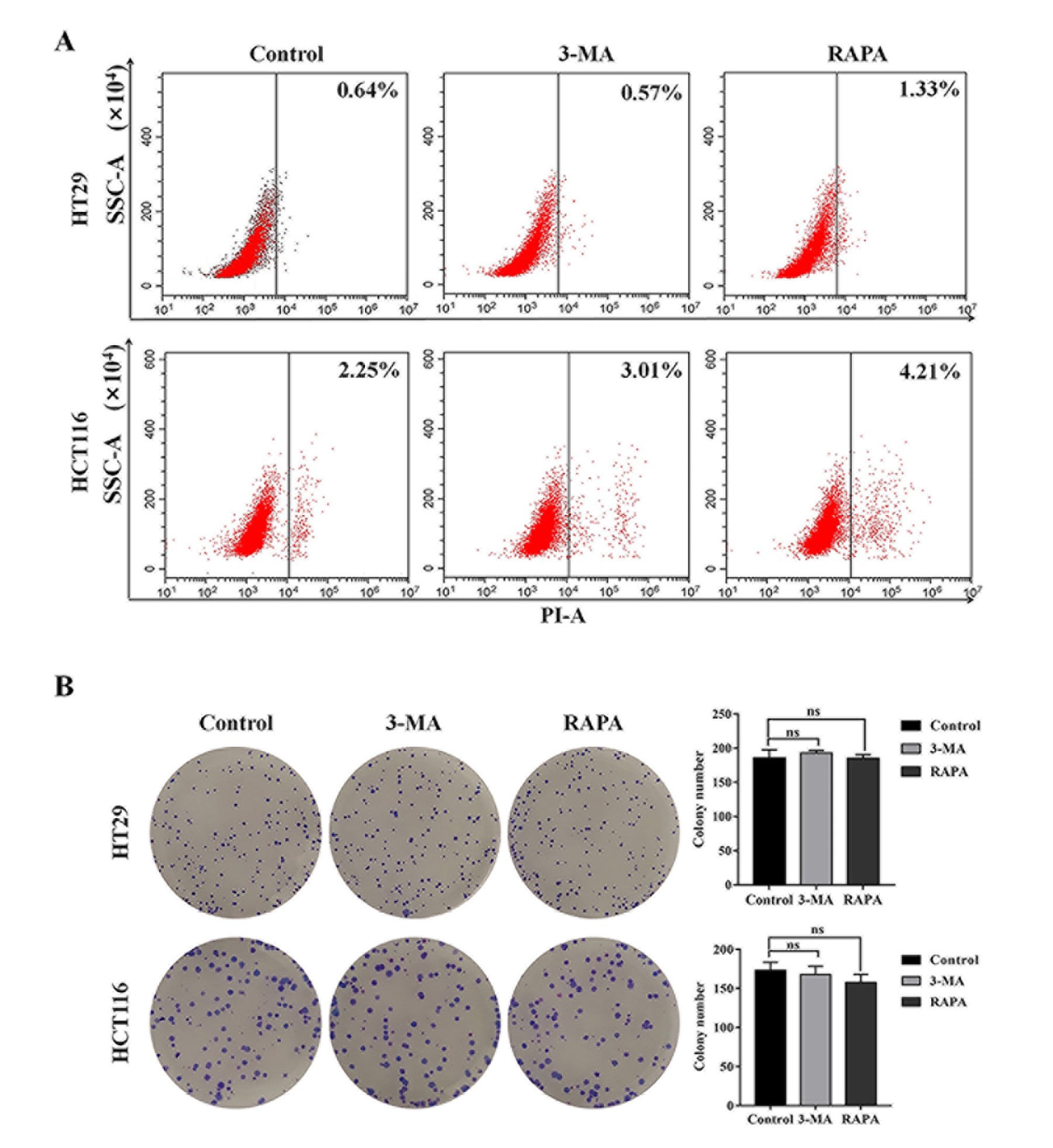

Supplement: Supplementary file 3 — Fig S3 [file JCMM-25-2110-s003.tif]

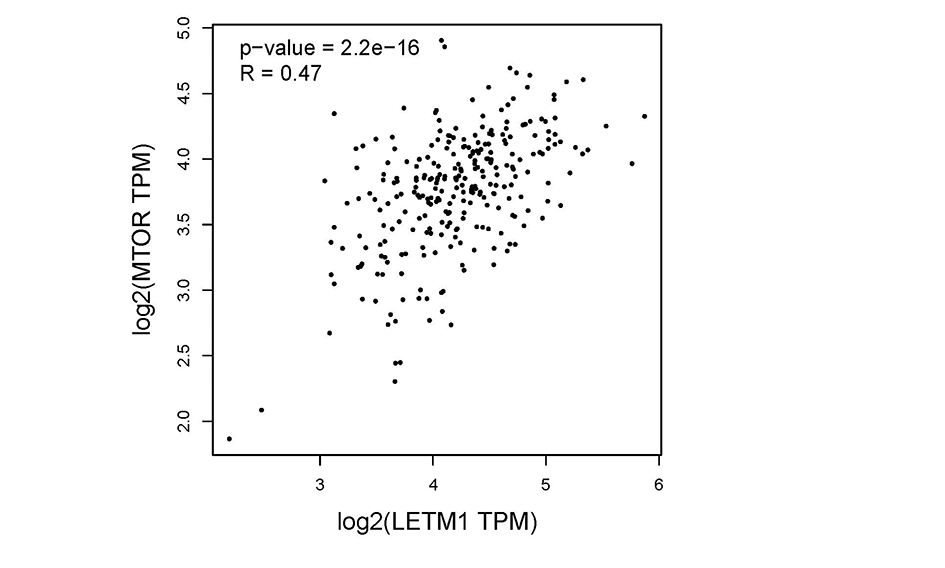

Supplement: Supplementary file 4 — Fig S4 [file JCMM-25-2110-s004.tif]

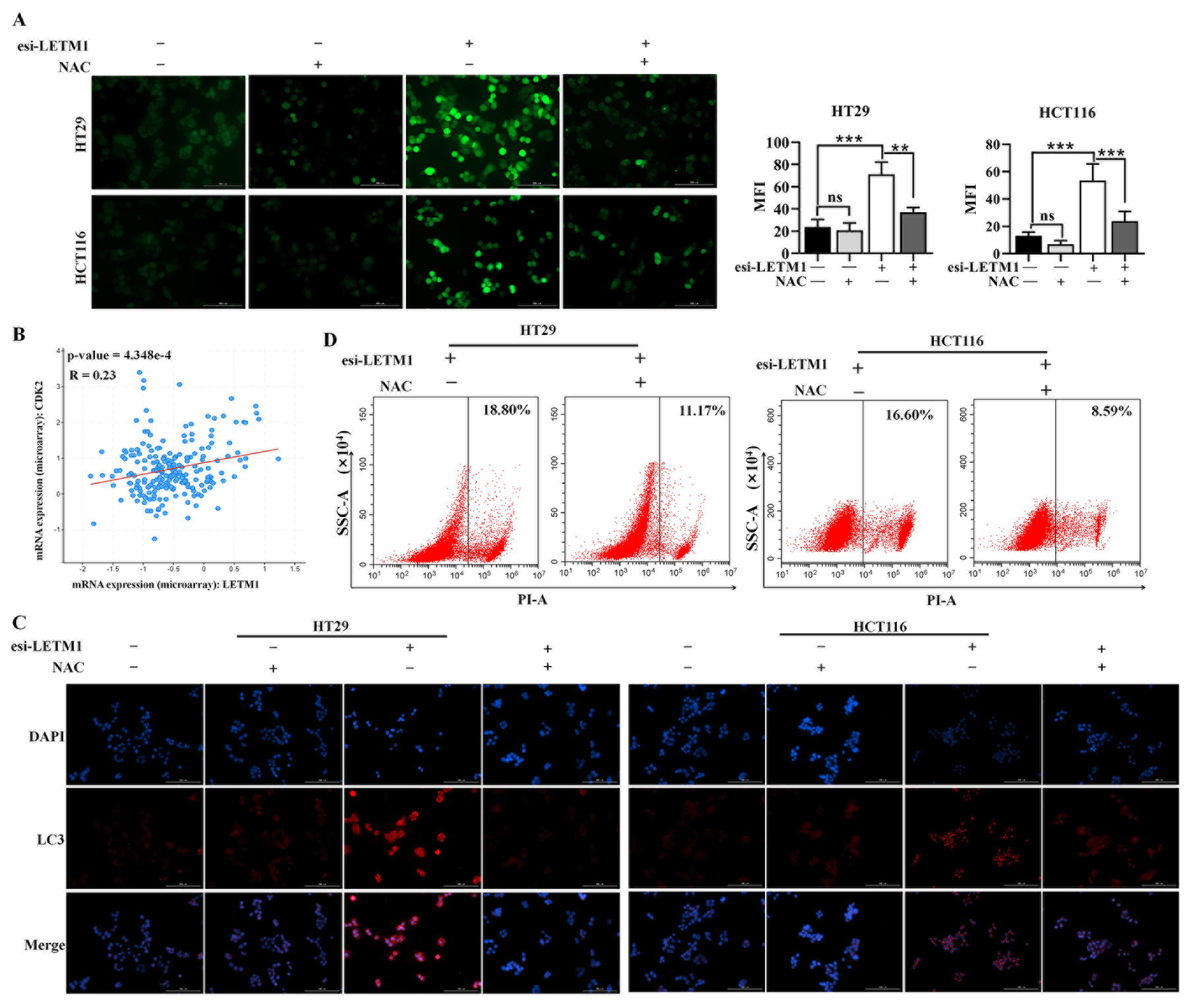

Supplement: Supplementary file 5 — Fig S5 [file JCMM-25-2110-s005.tif]
