# Supplementary material for: Suppression of LETM1 inhibits the proliferation and stemness of colorectal cancer cells through reactive oxygen species–induced autophagy
Source: J Cell Mol Med. 2020 Dec 13;25(4):2110–20. doi: 10.1111/jcmm.16169 (PMC7882971; doi:10.1111/jcmm.16169)
Supplement: Supplementary file 6 — Tab S1‐S2 [file JCMM-25-2110-s006.docx]

**Supplementary table 1. The sequence of LETM1 esiRNA.**

| **Gene** | **Sequence** |  |
| --- | --- | --- |
| LETM1 | | GTTCGCGATGACTCGGTAGTAGAGAAGTCCCTCAAGTCCTTGAAGGACAAGAACAAGAAGCTGGAGGAAGGCGGCCCGGTGTACAGCCCCCCCGCAGAGGTGGTGGTGAAGAAGTCCCTGGGGCAGCGGGTGCTGGACGAGCTGAAGCACTACTACCATGGCTTCCGCCTGCTATGGATCGACACCAAGATCGCGGCACGCATGCTCTGGCGCATCCTCAACGGCCACAGCCTGACCCGCCGGGAGCGCAGGCAGTTTCTCCGGATCTGCGCTGACCTCTTCCGCCTGGTGCCGTTCCTTGTGTTCGTGGTGGTGCCGTTCATGGAGTTTCTGCTGCCTGTTGCTGTGAAGCTCTTCCCCAACATGTTGCCATCCACATTTGAGACTCAGTCACTCAAGGAGGAGAGGCTGAAGAAGGAGCTTCGGGTCAAGCTGGAGCTGGCCAAGTTCCTCCAGGACACCATCGAGGAGAT. |

**Supplementary table 2. Antibodies in this study.**

| **Antibodies against** | **Company** | **Catalog number** |
| --- | --- | --- |
| LETM1 | Abcam | ab236743 |
| CD133 | Abcam | ab19898 |
| CD44 | Abcam | ab157107 |
| LC3B | CST | 2775 |
| Beclin 1 | CST | 3738 |
| Phospho-AMPKα-Thr172 | CST | 2531 |
| AMPKα | CST | 2532 |
| Phospho-mTOR-Ser2448 | CST | 2971 |
| mTOR | CST | 2972 |
| SOD2 | Abcam | ab13533 |
| CyclinA2 | CST | E1D9T |
| CDK2 | CST | 78B2 |
| β-actin | CST | D6A8 |
| GAPDH | CST | 14C10 |
